# Health care provider's perceived factors for the increased practice of caesarean delivery in North West Amhara referral hospitals, Ethiopia, 2022: a qualitative study

**DOI:** 10.3389/fgwh.2025.1401710

**Published:** 2025-02-03

**Authors:** Mulat Ayele, Marta Berta, Amare Zewudie, Eyob Shitie Lake, Gizachew Yilak, Befkad Derese Tilahun, Mastewal Belayneh Aklil

**Affiliations:** ^1^ Department of Midwifery, Collage of Health Science, Woldia University, Woldia, Ethiopia; ^2^ Department of Women’s and Family Health, School of Midwifery, University of Gondar, Gondar, Ethiopia; ^3^ Department of Public Health, Collage of Health Science and Medical School, Wolkite University, Wolkite, Ethiopia; ^4^ Department of Nursing, Collage of Health Science, Woldia University, Woldia, Ethiopia; ^5^ Department of Clinical Midwifery, School of Midwifery, University of Gondar, Gondar, Ethiopia

**Keywords:** caesarean delivery, caesarean section, qualitative study, health care providers, Ethiopia

## Abstract

**Background:**

Caesarean delivery is an essential obstetric intervention to reduce maternal and newborn mortality in emergencies. However, in Ethiopia, there is a high prevalence of caesarean deliveries. Therefore, this study aimed to explore the factors perceived by healthcare providers that contribute to the excessive rates of caesarean deliveries in North West Amhara referral hospitals, Ethiopia, in 2022.

**Methods:**

A phenomenological study design was employed, utilizing semi-structured interview guide for data collection. Fifteen healthcare providers working in referral hospitals in the north-western region of Amhara were interviewed using a heterogeneous purposive sampling approach until data was saturated. Transcribed interviews were translated coded and finally thematic analyses were employed using Open Code 4.0 software.

**Results:**

Healthcare providers observed a significant increase in the frequency of caesarean deliveries. Multiple factors were identified as contributing to this rise, including the involvement of medical students, the use of cardiotocography, a decline in instrumental deliveries, inadequate trial of labor after previous caesarean deliveries, and the absence of clear indications for performing caesarean deliveries for social or maternal requests. Notably, patients who had received care in private clinics were more likely to undergo caesarean deliveries.

**Conclusion:**

Caesarean deliveries were observed to be performed based on subjective or approximate indications, rather than clear obstetric indications. Encouraging greater emphasis on trial of labor, instrumental delivery, and performing caesarean deliveries only when there are definitive obstetric indications, rather than for social or maternal requests, can contribute to reducing the prevalence of caesarean delivery rates.

## Introduction

Caesarean delivery (CD) refers to the delivery of a viable fetus, placenta, and membrane through an incision in the abdominal wall and uterus (1). Access to caesarean deliveries can reduce inadequate obstetric outcomes such as maternal mortality, stillbirth, newborn death, obstetric fistula, uterine prolapse, and sexual dissatisfaction (2). However, women who undergo unnecessary caesarean delivery face risks such as severe maternal morbidity, hemorrhage requiring hysterectomy or transfusion, uterine rupture, anesthetic complications, shock, cardiac arrest, acute renal failure, assisted ventilation, venous thromboembolism, surgical site infections, and wound disruption or hematoma. The long-term risks include an increased likelihood of placenta previa and morbidly adherent placenta in future pregnancies, compared to vaginal delivery. For instance the risk of placenta previa for future pregnancy was increases from 1% with one prior CD to 3% with three or more previous CD. Additionally, the risk of morbidly adherent placenta were increased from 3% with no prior CD scar to 11%, 40% and 61% with one, two and three prior CD scar accompanied with placenta previa respectively (1, 3, 4). Caesarean delivery is associated with more complications compared to vaginal delivery, occurring 6–10 times more frequently (5, 6).

Although there are different relative and absolute indications for cesarean delivery, more than 85% of cesarean deliveries were performed for the indication of prior cesarean delivery, fetal jeopardy or non-reassuring fetal heart rate pattern, arrest of labor and fetal malpresentation (1). Half of the increase in the cesarean rate can be attributed to primary cesarean births. Within the primary cesarean category, subjective reasons such as a non-reassuring fetal heart rate pattern, suspected macrosomia, preeclampsia or eclampsia, multiple gestation, maternal request and arrest of cervical dilation played a major role compared to objective reasons such as malpresentation and maternal-fetal and obstetric conditions (7). In general, this is a reflection of poor obstetric practice. The decline in the trend of vaginal birth after cesarean section (VBAC) can be attributed to revised policy statements by organizations such as the American College of Obstetricians and Gynecologists (ACOG). These policy changes, which have been partially moderated in recent years, have made it increasingly challenging for medical institutions to offer VBACs due to concerns regarding liability (8). In addition to this, non-clinical factors are equally important factors for the rise of caesarean delivery. Economic incentives, free medical resources, health care providers' opinions, informed consent, private health care setting, lack of supervision and training in public hospitals, absence of or lack of familiarity with clinical guidelines, day time delivery, liability, differences in health provider practices, fear of malpractice litigation and organizational, economic, social and cultural factors are also contributing to the increasing incidence of cesarean deliver (8–12). In light of this, the American College of Obstetricians recommends various measures to reduce the primary caesarean delivery rate, such as redefining labor dystocia, improving fetal heart rate interpretation, providing continuous labor support, attempting external cephalic version for breech presentation, allowing trial of labor for twin pregnancies with the first twin in cephalic presentation, and considering instrumental vaginal delivery during the second stage of labor (3). Moreover, to tackle the escalating global rates of cesarean sections and mitigate the potential harm inflicted upon women and newborns due to excessive utilization of this procedure, the World Health Organization (WHO) published new recommendations on nonclinical interventions to reduce un necessary CD in 2018. The intervention guideline has different targets; intervention target women (for instance, childbirth training workshops, psycho-education), health-care providers target (implementation of evidence-based clinical practice guidelines, caesarean section audits and timely feedback to health-care professionals) and health organizations or facilities targets (different payment systems for caesarean sections, collaborative midwifery-obstetrician model of care) (13).

Even though the World Health Organization recommends those non clinical interventions to achieve the population level caesarean delivery in between 10 and 15% (14), the global rate of caesarean births is increasing significantly in different regions. In Western Europe, there has been a 24.5% increase, while in North America, the increase is 32%, and in South America, it is 41% (5). In Africa, caesarean delivery rates vary widely, ranging from 3% in Burkina Faso to 15.6% in Ghana (15).

In Ethiopia, aligning with the targets of Sustainable Development Goals 3 (SDG3), the Ethiopian government has made a steadfast commitment to enhance the availability of essential and comprehensive emergency care services. This dedication aims to diminish the Maternal Mortality Ratio (MMR) and neonatal mortality rates. Nonetheless, the pace of progress remains sluggish. According to the recent Mini Ethiopian Demographic Health Survey of 2019, a significant portion (51%) of childbirths continue to transpire within the home setting, predominantly facilitated by unskilled birth attendants (16). Despite poor progress in achieving high skilled birth attendance rate, the CD rate increases rapidly and varies across regions and over time, ranging from 0.4% in Somali to 21.4% in Addis Ababa, with a national pooled prevalence of 2% in 2016 and 29.5% in 2020 (4, 17–19). Similarly, the CD rate in the Amhara region ranged from 2.3% in 2016 (4) to 30.9% in 2020 (20). Variations and the increment in CD rates within and between hospitals can be attributed to intrinsic differences in hospital factors, infrastructure, the obstetric population served, and variations in clinical management protocols among physicians (20–22). Despite numerous quantitative studies on caesarean delivery rates and factors, the rate of CD continues to rise. Exploring the health care provider's perception towards the increasing rate of caesarean delivery might be an effective way to guide policy in reducing unnecessary CD. Therefore, this study aims to explore the factors perceived by healthcare providers that contribute to the high rate of caesarean deliveries in the study area using qualitative methods.

## Methods

### Study design, period and setting

**Figure 1:**
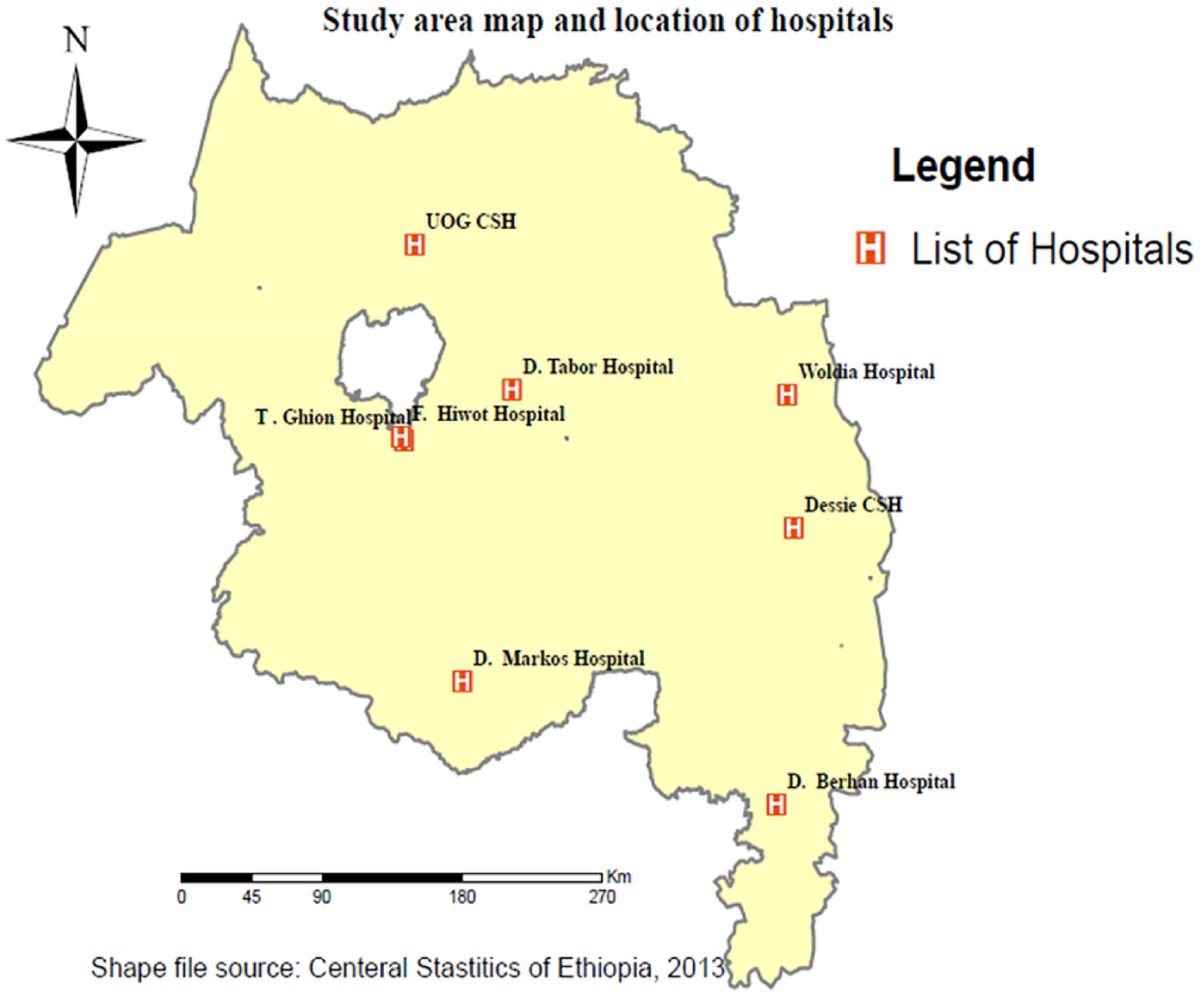
Study area map and location of Amhara regional comprehensive specialized public hospitals.

A phenomenological study design was conducted in three referral hospitals located in the North West Amhara region between January 10 and February 2, 2022. The Amhara regional state is one of the eleven regional states in Ethiopia, situated in the North West part of the country. Within this region, the North Western part comprises six zones with a total population of 13,049,742, of which 6,501,156 are females (23). There are eight public comprehensive specialized hospitals in Amhara region. Among this five were located in the North West part of the Amhara region, namely Debre Markos comprehensive specialized hospital, Felegehiwote comprehensive specialized hospital, Tibebe-Gihon comprehensive specialized hospital, Debre Tabor comprehensive specialized hospital, and the University of Gondar comprehensive specialized hospital (UOG CSH). However, this study focused only on Debre Markos, Debre Tabor, and Tibebe-Gihon comprehensive and specialized hospitals (Figure 1). Each of these hospitals serves a catchment population estimated to be between five and seven million people (23). According to recent reports from the hospitals' monthly records, the average number of mothers who give birth per month ranges from 600 to 700 for each referral hospital.

### Recruitment of study participants

The study involved healthcare providers employed in three specialized referral hospitals. A purposive sampling method was employed to select healthcare providers actively working in the labor and delivery units of each hospital. Fifteen healthcare providers participated in the study, including four senior obstetricians and gynecologists, four integrated emergency surgery and obstetrics (IESO) specialists, one senior clinical midwife, and six BSc midwives.

### Data collection process

Initially, the principal investigator developed an interview guide to ensure consistent coverage of the topics in each interview. An invitation letter was sent to the participants to fix the date and time of the interview.

Upon obtaining written informed consent, face-to-face interviews were conducted by the investigator at the participants’ workplaces. The interviews aimed to explore the participants' perceptions of caesarean delivery practices and factors. To establish a rapport with the participants, the interviews began with general open-ended questions about socio-demographic information. Following this, a set of questions related to the factors, and practices of caesarean delivery were presented. Based on each individual's response, additional open-ended questions were asked to further delve into their perspectives. Data collection concluded after fifteen individual interviews, as no new or unique information emerged (24). During the interviews, audio recording was used, and notes were taken to ensure accurate documentation. The interviews typically lasted between 30 and 45 min.

### Sampling procedure and technique

From the five referral hospitals in the North West Amhara region, a purposive selection was made to include only three hospitals in the study. To ensure a diverse representation, a heterogeneous purposive sampling technique was employed to select healthcare providers from each of the three selected hospitals.

### Positioning

Many researchers, particularly clinical midwives, possess extensive experience and knowledge related to the topic of interest, such as cesarean delivery. This positioning serves the purpose of contextualizing the research within a broader framework. It allows us to elucidate how our personal and professional backgrounds contribute to our understanding of the research topic and inform our approach to data collection and analysis. Moreover, positioning aids in presenting the findings in a manner that is easily comprehensible and devoid of bias, ensuring clarity for readers and the scientific community. By embracing positioning, we are prompted to critically examine our own biases, assumptions, and values and how these factors may influence every aspect of the research process, including data collection, analysis, and interpretation.

### Trustworthiness

To enhance the trustworthiness of the data, several strategies were employed. To ensure the credibility of the study, we used strategies such as triangulation involving the use of multiple data sources (from midwives, clinical midwives, IESOs and senior obstetricians) and researchers with different levels of experience and backgrounds to validate and corroborate the findings. We extended invitations to some participants to evaluate the study's findings and ideas, ensuring that their perspectives were accurately reflected. To enhance interobserver reliability, agreement measures were established to ensure consistency in the coding and interpretation of data between researchers. Peer debriefing and audit trials were also used to ensure the dependability of the qualitative study. Additionally, confirmability was addressed through reflexivity, peer review and transparent reporting.

Furthermore, the assessment of the transferability of data to a different set of circumstances relies on the contextual details provided by the researchers. Therefore, in this study, we included a comprehensive description of texts that aids readers in comprehending the surrounding conditions.

### Data management and analysis

Thematic data analysis was employed to analyze the data. Initially, the collected data was organized separately, and themes were generated to delve into rich details that aligned with the study's objective. To facilitate this process, all recorded data from the in-depth interviews underwent transcription, converting spoken words into written text. Furthermore, language experts translated the transcriptions into English to enhance clarity and comprehension. Next, we meticulously read through the transcriptions multiple times, applying coding techniques using open code software version 4.0. The coding process involved labeling and categorizing segments of the text that shared similar concepts or ideas. By organizing these codes, synthesis was conducted to identify and articulate overarching themes. This enabled us to grasp and convey the general idea and ultimately achieve the study's objective. Finally, the findings were reported by writing a descriptive account of the text and providing interpretations of the identified themes. Direct quotes were included to offer vivid examples for readers and enhance the trustworthiness of the findings. This comprehensive approach aimed to present a clear understanding of the data analysis process and communicate the meanings derived from the themes.

### Ethical consideration

The study received ethical approval from the Institutional Review Board (IRB) of the University of Gondar under reference number Ref.N/53/2014E.C. Participants were provided with a clear explanation of the study's objectives and assured of the confidentiality of their data. Informed written consent was obtained from all participants. Throughout the study, all methods adhered to the applicable guidelines and regulations outlined in the Declaration of Helsinki.

## Results

In-depth interviews were conducted with fifteen healthcare providers involved in maternal and newborn care, with each interview lasting approximately 30–45 min. These interviews aimed to gather qualitative data regarding factors related to CD. The majority of participants (6 out of 15) were midwives, followed by senior obstetricians and gynecologists (4 out of 15), one senior clinical midwife, and four senior integrated emergency surgery and obstetrics (IESO) specialists from the three selected hospitals. The interviews were recorded, and the data was transcribed and summarized based on emerging themes. During the analysis process, two main themes emerged: healthcare providers’ practices and their perceived factors influencing caesarean delivery.

### Theme 1: health care providers' obstetrical practices that increase caesarean delivery;

Healthcare providers employ various practices that influence the rate of caesarean deliveries. These practices encompass instrumental delivery, trial of labor after caesarean delivery (TOLAC), social or relative factors, caesarean delivery by maternal request (CDMR), and the consideration of primigravida with breech presentation as an indication for caesarean delivery.

### Health care providers practice on caesarean delivery by maternal request (CDMR);

Although CDMR is not permitted in the country, many obstetricians and gynecologists still carry it out, particularly those who work in private clinics or both private and government hospitals. They shift the indication from false to true, using reasons such as oligohydramnios, primigravida with breech presentation, non-reassuring fetal heart rate pattern (NRFHRP), and others.

One participant explained, “*Most obstetricians and gynecologists perform caesarean delivery by maternal request by changing the indication to a valid one. For example, they justify CDMR by citing oligohydramnios, primigravida with breech presentation, NRFHRP, and so on.”*

Another participant mentioned, “*In this hospital, caesarean delivery by maternal request is mostly done for staff, mothers with social connections, and mothers receiving antenatal care in private clinics.”*

### Healthcare providers' practice of instrumental delivery

Instrumental delivery, such as forceps, is rarely practiced in most hospitals due to concerns about side effects and a lack of skill among providers, including obstetricians and gynecologists. As a result, they prefer to perform caesarean deliveries instead of using forceps or vacuum. Almost all healthcare providers in most hospitals do not practice instrumental delivery, especially forceps. Participant 7 stated, “*In this hospital, instrumental delivery is almost never performed due to the potential side effects associated with it.”*

### Healthcare providers' practice in private clinics and with social or relatives

Obstetricians and gynecologists who work in both government and private clinics sometimes perform caesarean deliveries without a valid indication or with an approximate one. This is because those with private clinics have their own clientele and prioritize their clients' requests or interests to promote and develop their businesses. Consequently, they perform caesarean deliveries without a proper indication, but falsely document a justification for the procedure.

Participant 1 explained, “*I am confident that some obstetricians with private clinics use caesarean delivery as a promotional tool, fulfilling the clients’ interests and thinking that it boosts their business.”*

Participant 6 also noted, “*In this hospital, caesarean deliveries are performed for social reasons, but the indication is falsely documented as something other than social or related factors, such as NRFHRP.”*

### Healthcare providers' practice of trial of labor after caesarean delivery (TOLAC)

In most hospitals, the practice of TOLAC is often inadequate due to insufficient counseling, the desire to avoid accountability, heavy workload, and negligence. Some providers steer cases toward caesarean delivery by presenting women with a choice between CD and TOLAC, as women with a previous caesarean scar require more attention. Although some providers attempt to promote TOLAC, women often decline it due to fear of complications and labor pain, opting for repeated caesarean deliveries.

“*In our hospital, most staff tend to direct women towards caesarean delivery if they have a previous caesarean scar due to fears of close follow-up and accountability for women at risk of uterine dehiscence,”* shared participant 8.

### Healthcare providers' practice regarding primigravida women with breech presentations

The acceptance of term breech trials varies among different obstetricians, but nearly all obstetricians and gynecologists, especially those with private clinics, currently consider primigravida with breech presentation as a direct indication for caesarean delivery. This practice aims to reduce neonatal mortality. In most hospitals, caesarean delivery is commonly performed for primigravida with breech presentation based on the belief that it prevents complications related to the baby's head descending during delivery.

Participant 3 explained, “In this hospital, experienced obstetricians and gynecologists consider primigravida with breech presentation as a direct indication for caesarean delivery because they believe it prevents complications associated with the after coming head.”

In contrast, participant 6 mentioned, “*In this hospital, the mode of delivery for primigravida with breech presentation is determined based on the women's decision after brief counseling about the advantages, disadvantages, and available delivery options.”*

### Theme 2: perceived factors of caesarean delivery

Healthcare providers or participants identified numerous factors that were perceived to contribute to the increase in caesarean delivery rates.

### Case misdiagnosis or a lack of early detection and intervention

Delayed care-seeking by pregnant women often resulted in obstetric complications, such as uterine rupture, cephalopelvic disproportion (CPD), unnecessary caesarean delivery, hysterectomy, and maternal and fetal mortality. Participants believed that early detection and timely intervention could prevent complications and unnecessary caesarean deliveries.

“*Women waiting in healthcare facilities with inadequate infrastructure, such as health centers or primary hospitals, without timely detection and intervention were more likely to experience complications like obstructed labor and require referrals.”* participant 3 stated.

Diagnosis problems, especially among students and some cardiotocography (CTG) staff were identified as another factor contributing to incorrect decisions and the increase in unnecessary caesarean deliveries.

“*Currently, most of the indications for CD are NRFHRP. This may be due to a diagnosis problem or may be a diagnosis by less experienced staff*.” Participant 1 said.

### The presence of medical students in labor and delivery ward

The presence of medical students in the labor and delivery ward was perceived by almost all participants as a factor that contributes to an increase in the caesarean delivery rate. This increase was attributed to two main reasons: fear of accountability from obstetricians and gynecologists and the students' interest in practicing the skill of caesarean delivery.

Participants noted that most medical students, including interns, IESOs, clinical midwives, and residents, lacked experience and knowledge in various aspects related to childbirth, such as reading and interpreting cardiotocography machine readings, understanding the properties of older CTG machines, and implementing supportive measures like resuscitation, oxygenation, or using fetoscopes. Instead, these students tended to rely on a single episode of fetal heart rate (FHR) reading and based their conclusions solely on that, without considering the overall FHR pattern. This overreliance on non-reassuring fetal heart rate patterns (NRFHRP) often led to the indication for caesarean delivery, contributing to the increased rate. One participant explained that;

“*When medical students encounter an NRFHRP during their observations of the mother, some students consult the obstetricians early due to fear and frustration associated with being students. These students may not give enough time or consider interventions to address the NRFHRP but quickly consult the obstetricians, who then accept or decide based on that consultation without thoroughly evaluating whether the NRFHRP is genuinely non-reassuring or if it can be corrected with interventions. The participant further mentioned that this situation is more common among interns and less likely among clinical midwives, suggesting that experience plays a role in student decision-making.”*

Additionally, some students, particularly IESOs, may influence the decision for caesarean delivery by approximating indications and presenting them as absolute indications, even in cases that are not complicated or require immediate intervention. These students aim to gain experience in the skill of caesarean delivery and consult seniors, who may approve the procedure without thoroughly assessing the situation. This practice further contributes to the increase in the caesarean delivery rate.

“*Students, especially IESO students, want to do, learn, or know the skill of caesarean delivery. So, they approximate the indication and consult for the senior, telling the relative indication as an absolute indication and the cold case as a complicated case, and the senior said prepare for CD without checking, and they perform caesarean delivery. So, this increases the CD rate.”* Participant 1 said.

### Lack of skill or fear of side effects of using instrumental delivery

The lack of proficiency or concerns regarding the potential side effects associated with instrumental delivery is a contributing factor to the rise in caesarean delivery rates. Healthcare providers, including obstetricians and gynecologists, may exhibit a fear of complications such as neonatal issues, maternal complications, and genital tears, which subsequently leads to an increased preference for caesarean delivery over instrumental delivery. Furthermore, the inadequate skill in correctly utilizing instruments, particularly forceps, by these providers further contributes to the higher rate of caesarean deliveries.

Participant 2 provided an explanation, “*stating that when a staff member encounters a prolonged second stage of labor, they are often hesitant to opt for instrumental delivery due to apprehension about potential complications and the fear of being held accountable. In such situations, healthcare professionals may prioritize caesarean delivery as a safer alternative, driven by concerns related to accountability and the perceived side effects associated with instrumental delivery.”*

In summary, the lack of proficiency in instrumental delivery techniques and the fear of adverse effects are factors that contribute to the increased utilization of caesarean delivery over instrumental delivery.

### Decreased vaginal birth after caesarean delivery (VBAC) or trial of labor after caesarean delivery (TOLAC)

The reduction in VBAC or TOLAC was perceived by most participants as a factor that contributes to the increased caesarean delivery rate among women with previous caesarean delivery scars. While decreasing primary caesarean delivery is a primary strategy to reduce the overall caesarean delivery rate, encouraging VBAC or TOLAC is another approach. However, inadequate counseling regarding the advantages and disadvantages of TOLAC and repeated caesarean delivery, as well as inadequate explanation of the meaning of consent and the potential future complications associated with TOLAC and repeated caesarean delivery, contribute to the increased rate of repeated caesarean deliveries. This lack of comprehensive counseling is observed in both government and private clinics.

Participant 7 explained that “the success of VBAC or TOLAC depends on the quality of counseling provided. During antenatal care follow-up, if healthcare providers briefly discuss the risks, benefits, and anticipated complications of TOLAC and repeated caesarean delivery with women, tailoring the information to their level of education, women are more likely to consider TOLAC. However, if counseling lacks detailed information about the benefits of TOLAC and the percentage of complications, especially for women coming from rural areas, they may only hear about the potential complications and decline TOLAC.”

Participant 8 also mentioned that “*poor counseling for TOLAC, both in government and private clinics, particularly among obstetricians and gynecologists working in private clinics, contributes significantly to the preference for caesarean delivery over TOLAC. Inadequate counseling practices have played a substantial role in the increasing trend of caesarean deliveries compared to the past.”*

Furthermore, some providers hold the perception that midwives tend to push women with previous caesarean delivery scars towards repeated caesarean delivery. This perception arises from the fear of the additional close follow-up required for mothers attempting TOLAC, as women with previous caesarean delivery scars often need more diligent monitoring. Consequently, certain providers may counsel women towards caesarean delivery during admission to avoid the challenges associated with strict follow-up.

“*Mother who has previous CD scar needs more proper follow up. However, in our hospital due to fear of this follow up most staffs push such women towards CD.”* Participant 8 explained.

### Poor knowledge of cardiotocography (CTG) and its interpretation

Insufficient knowledge regarding CTG and its interpretation is perceived by most healthcare providers as a contributing factor to the increased caesarean delivery rate. This lack of knowledge leads to the inappropriate use of CTG, with decisions being based solely on the CTG number without considering its properties. Despite the recommendation for continuous intrapartum follow-up using CTG, providers often lack comprehensive understanding of the machine's properties, operation, and how to interpret the CTG tracings. Consequently, many providers solely rely on the CTG record number to diagnose non-reassuring fetal heart rate patterns (NRFHRP), regardless of whether that number accurately reflects the fetal heart rate (FHR). Additionally, most CTG machines do not print the complete FHR pattern but only consider short patterns. Moreover, older CTG machines may produce false FHR readings even in cases of intrauterine fetal death or without proper connection to the maternal abdomen. These factors collectively contribute to an increased rate of caesarean deliveries based on the indication of NRFHRP.

Participant 4 best explained the issue by highlighting that *“while continuous fetal monitoring is recommended, the problem lies in the inadequate training on the proper usage, operation, and interpretation of CTG results, rather than simply relying on the machine itself. For instance, the participant pointed out that although there may be a fetal heartbeat tracing on the CTG, healthcare providers may lack the necessary experience to accurately interpret it. Moreover, the CTG itself is highly sensitive, and factors such as maternal ambulation, changes in position, or incorrect placement of the CTG on the abdomen can result in erroneous readings. These incorrect readings could potentially influence the decision towards caesarean delivery, particularly if providers are unaware of these limitations.”*

In summary, the poor knowledge of cardiotocography and its interpretation contributes to the increased caesarean delivery rate. Inadequate training on how to properly use, operate, and interpret CTG results, along with the sensitivity of the CTG and the limitations of older machines, leads to decisions based on incomplete or inaccurate information. As a result, caesarean deliveries are often performed based on the indication of NRFHRP.

### The presence of social or relatives and the expansion of private clinics

The presence of social factors or relatives, particularly in private clinics, is widely perceived by healthcare providers as the primary factor contributing to the increase in CD rates. Obstetricians working in private clinics, driven by business considerations and the desire to satisfy their clients, tend to perform CDs for women who receive ANC follow-up in their clinics, even without clear medical indications. This practice involves providing undiagnosed indications for CD to gain social acceptance and accommodate the preferences of their clients.

Participant 2 explained that “the current rise in CD rates is largely influenced by social factors. When a mother receives ANC follow-up in a private clinic, the obstetricians working there may perform unjustified CDs by providing undisclosed indications, solely to gain social acceptance within that hospital.”

### Number of seniors or surgeons

Most participants believed that an increase in their numbers does not directly lead to an increase in the overall CD rate, especially in emergency cases. However, they acknowledged that the number of seniors may impact the CD rate in elective cases, particularly in social or private clinics. In emergency situations, if a mother is a candidate for CD due to complications, it becomes necessary regardless of the number of seniors available.

Participant 7 expressed this by stating that “*CD rates are not affected by the increment of seniors, but the number of seniors may have an influence on elective cases, especially in social or private clinics.”*

Contrarily, some providers believe that an increase in the number of seniors or surgeons does contribute to the higher CD rate. This perspective arises from the fact that different seniors have varying references, experiences, and practices acquired from different universities or hospitals. For example, one senior may consider primigravida with breech presentation as an indication for CD, while another senior may argue that it is not a valid indication unless scientifically justified. Participant 1 illustrated this by highlighting that “*different seniors come with different indications and experiences based on their training institutions.”*

Other perceived factors contributing to the increased CD rate include poor adherence to the use of the partograph, limited community awareness regarding the complications of CD, variations in senior experience, skill gaps among midwives, community acceptance or adaptation of CD, the practice of CD for primigravida with breech presentation, fear of labor pain, and the workload of seniors.

## Discussion

This study provided valuable insights into the perceived factors and practices surrounding caesarean delivery. Instead of being performed solely based on absolute medical indications, caesarean deliveries were found to be influenced by various factors and relative indications such as caesarean delivery on maternal request (CDMR), social or family influences, and primigravida with breech presentation. Although caesarean delivery by maternal request is not permitted in our country, the results of this study indicated that it was still being performed based on this indication. These findings are consistent with previous studies (25–27) and align with research conducted in Argentina (25) which also reported caesarean deliveries being performed for social or family-related reasons without obstetric indications.

According to established guidelines, indications for caesarean delivery in cases of breech presentation include estimated fetal weight less than 1,500 g or greater than 4,000 g, hyperextended head, and footling breech (1). However, in this study, most healthcare providers in all hospitals acknowledged that, as the term breech trial approaches, breech presentation is considered an indication for caesarean delivery by most senior professionals, especially in primigravida mothers. This finding is supported by a study conducted in Iran (28). The study provided valuable insights into the healthcare providers' deviations from following the scientifically established indications for caesarean delivery. Identifying the barriers that prevent the adherence to absolute or scientific indications for caesarean delivery would be beneficial for leaders and healthcare providers in promoting adherence to these guidelines.

This study identified several factors perceived to contribute to the rise in caesarean deliveries. The presence of social or family members and the expansion of private clinics emerged as the most significant factors driving the increase. Particularly in private obstetrics and gynecology specialty clinics, social or familial factors played a major role in the increment of caesarean deliveries. This aligns with findings from previous studies, which reported that seniors working in both government and private clinics often performed caesarean deliveries for their clients without a medical indication based solely on maternal preference (28–30). Another factor identified in this study was the poor knowledge and interpretation of cardiotocography. Despite the recommendation for continuous intrapartum fetal monitoring for high risk pregnancies (31, 32), inadequate understanding of CTG and its interpretation led to inappropriate decisions and a higher rate of caesarean deliveries. The widespread use of electronic fetal monitoring, coupled with suboptimal CTG interpretation, was associated with an increased incidence of non-reassuring fetal heart rate patterns, leading to unnecessary caesarean deliveries. This is supported by different studies (33–35). Therefore, it is important for healthcare providers to understand the fundamental patterns of fetal heart rate that indicate concern and interpret them correctly. This knowledge can help decrease the rate of unnecessary caesarean deliveries. One effective non-clinical measure to reduce caesarean births is the implementation of evidence-based clinical practice guidelines. Additionally, it is recommended to have a structured and mandatory second opinion process for determining the need for caesarean sections. This approach is particularly beneficial in settings with sufficient resources and experienced senior clinicians who can provide the required second opinions, as recommended by the World Health Organization (13).

The presence of various medical students, including interns, clinical midwives, integrated emergency surgeons (IESO), and residents in labor and delivery wards, was identified as another contributing factor to the increase in caesarean delivery rates in this study, primarily due to the influence and frustration of their senior colleagues. Some students, like interns, made early decisions and sought consultation with their seniors, while others, such as IESO, clinical midwives, and residents, had a strong interest in practicing caesarean delivery skills. As a result, they sometimes misinterpreted indications and made inappropriate decisions, leading to unnecessary caesarean deliveries. A study conducted in Iran supports these findings (36).

Fear of the potential side effects associated with instrumental delivery, such as perineal tear and cephalohematoma, was identified as another contributing factor to the rising caesarean delivery rates. Many healthcare providers perceived that instrumental delivery, particularly forceps delivery, was not commonly practiced in all hospitals due to insufficient skills and concerns about complications. Consequently, the reduced use of instrumental delivery contributed to an increase in the overall caesarean delivery rate. Similar findings have been reported in studies conducted in different countries (1, 37). The number of senior professionals or surgeons was also perceived as a factor contributing to the rise in caesarean deliveries by the majority of participants. They believed that an increased number of seniors led to practice and reference variations, ultimately resulting in higher caesarean delivery rates. This observation is supported by studies conducted in the Wolayta Zone and Iran (38, 39). However, some participants held the view that the number of surgeons or seniors alone was not a significant factor in the increase of caesarean deliveries. Instead, they emphasized that the growth of private clinics and social factors associated with the number of seniors or surgeons played a more influential role (30).

In this study, the decrease in vaginal birth after caesarean delivery (VBAC) was identified as a significant contributing factor to the increase in the caesarean delivery rate. This decrease was attributed to the fact that women with previous caesarean delivery scars require strict and frequent monitoring. As a result, healthcare providers often push these women towards repeated caesarean deliveries instead of offering them a trial of labor, as the women themselves may be hesitant to attempt vaginal birth. A study conducted in Bangladesh supports these findings (30). Despite the recommendation by the American obstetrics and gynecology guidelines to allow women to undergo a trial of labor to reduce the caesarean delivery rate, the current trend shows a decline in VBAC rates and an increase in repeated caesarean deliveries (1, 3). This can be attributed to inadequate counseling provided to women about the risks and benefits of both repeated caesarean delivery and trial of labor, starting from antenatal care (39, 40). Thus, it is crucial for healthcare providers to adopt the non-clinical intervention recommendation from the World Health Organization (WHO) that targets on women. This recommendation involves conducting childbirth training workshops that cover various topics such as childbirth fear and pain, pharmacological pain-relief techniques and their consequences, non-pharmacological pain-relief methods, the pros and cons of caesarean sections vs. vaginal delivery, as well as indications and contraindications of caesarean sections, among other relevant subjects. The goal of these workshops is to reduce the rate of unnecessary caesarean deliveries (13).

### Strength and limitation of the study

As far as we know, this is the initial qualitative study conducted in Ethiopia to explore the factors perceived by healthcare providers that contribute to the rise in caesarean deliveries. Therefore, it provides valuable understanding regarding the practice and factors associated with caesarean deliveries by healthcare providers.

However, there are certain limitations to this study. Although data saturation was achieved early on, the ability to generalize or transfer the results from interviews with a small sample size to the larger population may be compromised. All interviews were conducted by the investigators, which could introduce some bias. However, to mitigate this, transcription was carried out by professional colleagues who possess extensive experience in qualitative research.

### Implication of the study

This study provides valuable insights into the factors contributing to the increased caesarean delivery rate and highlights the possible non-clinical interventions to reduce the unnecessary caesarean delivery, which supports the world health organization recommendation on non-clinical interventions to reduce unnecessary caesarean sections. It highlights the need for hospital managers to prioritize training on cardiotocography due to the demonstrated lack of knowledge among healthcare providers regarding CTG machines and their interpretation, which leads to an increased incidence of non-reassuring fetal heart rate pattern (NRFHRP) indicated caesarean deliveries. Additionally, the study sheds light on poor practices, such as inadequate trial of labor after cesarean (TOLAC) practices, inappropriate caesarean delivery for hospital staff, and a decline in instrumental delivery practices, calling for efforts to strengthen and improve these areas of care. Furthermore, the study raises an important question for future researchers, healthcare providers, and regional and federal health ministry's regarding caesarean deliveries performed upon maternal request, repeated caesarean delivery and breech presentation.

## Conclusion

This study revealed that caesarean deliveries were often performed based on relative indications or approximations, particularly for women with social or family connections and those receiving care in private clinics. The identified factors contributing to the increase in caesarean delivery rates included the presence of medical students, a growing number of senior professionals, the expansion of private clinics, inadequate knowledge or training on cardiotocography (CTG) and its interpretation, fear or lack of skill in instrumental delivery, reduced rates of vaginal birth after caesarean (VBAC) due to inadequate counseling and fear of complications, the influence of social or family members, and caesarean delivery on maternal request.

Based on these findings, we recommend that hospital managers and maternal and child health (MCH) coordinators focus on reviewing the practice of trial of labor and caesarean delivery, particularly in cases involving social or maternal requests, to prevent unjustified caesarean deliveries. It is crucial to provide training on the usage and interpretation of CTG for both medical students and healthcare staff. Obstetricians and other healthcare providers should actively promote the practice of VBAC through effective counseling in both government and private clinics.

## Data Availability

The original contributions presented in the study are included in the article/Supplementary Material, further inquiries can be directed to the corresponding author.
